# The impairment of small nerve fibers in severe sepsis and septic shock

**DOI:** 10.1186/s13054-016-1241-5

**Published:** 2016-03-15

**Authors:** Hubertus Axer, Alexander Grimm, Christine Pausch, Ulrike Teschner, Jan Zinke, Sven Eisenach, Sindy Beck, Orlando Guntinas-Lichius, Frank M. Brunkhorst, Otto W. Witte

**Affiliations:** Hans Berger Department of Neurology, Jena University Hospital, Erlanger Allee 101, D-07747 Jena, Germany; Department of Neurology and Epileptology, University of Tuebingen, Tuebingen, Germany; Center for Sepsis Control and Care (CSCC), Jena University Hospital, Jena, Germany; Institute for Medical Informatics, Statistics and Epidemiology (IMISE), University Leipzig, Leipzig, Germany; Department of Otorhinolaryngology, Jena University Hospital, Jena, Germany; Center for Clinical Studies, Jena University Hospital, Jena, Germany

**Keywords:** Sepsis, Critical illness polyneuropathy, Skin biopsy, Small nerve fiber neuropathy

## Abstract

**Background:**

A decrease of small nerve fibers in skin biopsies during the course of critical illness has been demonstrated recently. However, the diagnostic use of skin biopsies in sepsis and its time course is not known.

**Methods:**

Patients (n=32) with severe sepsis or septic shock were examined using skin biopsies, neurological examination, nerve conduction studies, and sympathetic skin response in the first week after onset of sepsis, 2 weeks and 4 months later and compared to gender- and age-matched healthy controls.

**Results:**

Skin biopsies at the ankle and thigh revealed a significant decrease of intraepidermal nerve fiber density (IENFD) during the first week of sepsis and 2 weeks later. All patients developed critical illness polyneuropathy (CIP) according to electrophysiological criteria and 11 showed IENFD values lower than the 0.05 quantile. Four patients were biopsied after 4 months and still showed decreased IENFD. Results of nerve conduction studies and IENFD did considerably change over time. No differences for survival time between patients with IEFND lower and larger than 3.5 fibers/mm were found.

**Conclusions:**

Skin biopsy is able to detect an impairment of small sensory nerve fibers early in the course of sepsis. However, it may not be suited as a prognostic parameter for survival.

**Trial registration:**

German Clinical Trials Register, DRKS-ID: DRKS00000642, 12/17/2010

**Electronic supplementary material:**

The online version of this article (doi:10.1186/s13054-016-1241-5) contains supplementary material, which is available to authorized users.

## Background

Severe sepsis and septic shock represent the third leading cause of death in industrialized countries [1, 2] and are among the most challenging conditions in modern medicine. A particularly harmful complication of sepsis is ICU-acquired weakness (ICUAW) [3, 4]. ICUAW may be caused by muscle wasting [5], critical illness polyneuropathy (CIP), critical illness myopathy (CIM), or as in most cases, a combination of CIP and CIM [6]. About 70 % of patients with sepsis [7], and up to 100 % of patients with multi-organ failure develop CIP [8], but this may be an overestimation due to use of different diagnostic criteria between studies [9]. Development of CIP is associated with increased mortality, prolonged times on the respirator, and longer rehabilitation periods [10–12].

CIP and CIM share the major clinical signs comprising symmetric and flaccid weakness of the muscles and the absence of deep tendon reflexes [13]. Patients with CIP also have distal loss of sensitivity to pain, temperature, and vibration. Nerve conduction studies detect CIP and CIM early during the first week of the disease [14, 15]. Typical signs of CIP and CIM are reduction in the amplitude of compound muscle action potentials, whilst nerve conduction velocity is mainly preserved. In CIP, amplitudes of sensory nerve action potential (SNAP) may also be reduced or missing, indicating axonal damage to sensory nerves [16, 17].

In recent years skin biopsy has become a valuable tool for the evaluation of patients with small nerve fiber pathological change [18, 19], with reliable normative data [20]. Skin biopsy obtained using a disposable 3-mm-diameter punch is minimally invasive and generally safe [21]. Skin biopsy is typically taken at the ankle and/or the thigh. The tissue is processed histologically with staining of intraepidermal nerve fibers. Nerve fiber density can be estimated by counting the nerve fibers crossing the basal lamina of the epidermis over the surface length of 50-μm-thick sections of the skin. Intraepidermal nerve fiber density (IENFD) closely correlates with clinical symptoms and is more sensitive than sensory nerve conduction studies and sural nerve biopsies for diagnosing small fiber neuropathy. Diagnostic efficiency and predictive values are very high using this technique [18, 22].

An impairment of small nerve fibers in sepsis may play a major pathophysiological role in neuropathic pain syndromes, which often become apparent after recovery from severe illness and after discharge from the ICU [23]. In addition, autonomic dysregulation plays a significant role in the acute phase of severe sepsis [24]. Lately, the occurrence of CIP has been related to decreased heart rate variability [25]. It is well-known that autonomic dysregulation is associated with increased long-term mortality rates [26, 27]. In addition, there appears to be correlation between IENFD and abnormal autonomic function assessed by quantitative sudomotor axonal reflex test in patients with painful neuropathy [28]. Therefore, we hypothesized that impairment of small nerve fibers plays a role as a prognostic marker in sepsis.

Latronico et al. [29] performed skin biopsies at the ankle and thigh in 14 adult patients under neurocritical care, who had a prolonged ICU length of stay and artificial ventilation on median ICU-day 22. All patients had severe and non-length-dependent loss of intraepidermal nerve fibers. Of these patients 13 had infections, sepsis or multiple organ failure. In addition, sweat gland innervation was reduced in all except one patient.

In another study [30], IENFD of the distal leg was assessed on admission to the ICU and 10–14 days later. Of the 11 patients recruited, 9 (82 %) had sepsis or multiple organ failure. Median IENFD on admission decreased significantly. Abnormal IENFD was observed in 8 patients (72.7 %). Electrodiagnostic signs of large fiber neuropathy and/or myopathy were found in 6 patients (54.5 %), and autonomic dysfunction in 2 patients (18.2 %).

The aim of this study was to examine the decrease in intraepidermal nerve fibers in CIP in the early phase of sepsis, to describe the time course of intraepidermal nerve fiber density at the ankle and thigh during the course of sepsis, and to evaluate the use of IENFD as a prognostic marker in the acute phase of sepsis.

## Methods

### Study design

The study protocol for this single-center, non-randomized, controlled, observational study has been published earlier in detail [31]. The study has been registered with the German Clinical Trials Register (DRKS-ID: DRKS00000642).

Patients ≥18 years of age with severe sepsis and/or septic shock according to published criteria [32] were screened for eligibility. Exclusion criteria were: history of neuromuscular disorders (such as polyneuropathy, myasthenia gravis, myopathy, and others), known alcohol abuse, high-dose steroid therapy before sepsis (≥16 mg prednisolone/kg body weight for 5 days or equivalent dose of other corticosteroids), ICU stay ≥8 days, participation in another clinical study, platelet count <40 Gpt/l(10^9^/l), partial thromboplastin time (PTT) >60 s, international normalized ratio (INR) >1.7, or if the patient was likely to die within less than 24 hours.

Patients admitted to the ICUs at Jena University Hospital between February 2011 and April 2015 were enrolled in the study. Written informed consent was obtained from all patients or their legal representatives and from all healthy controls. The study was approved by the Ethics Committee of the Friedrich Schiller University Jena (number 2771-02/10).

**Fig. 1:**
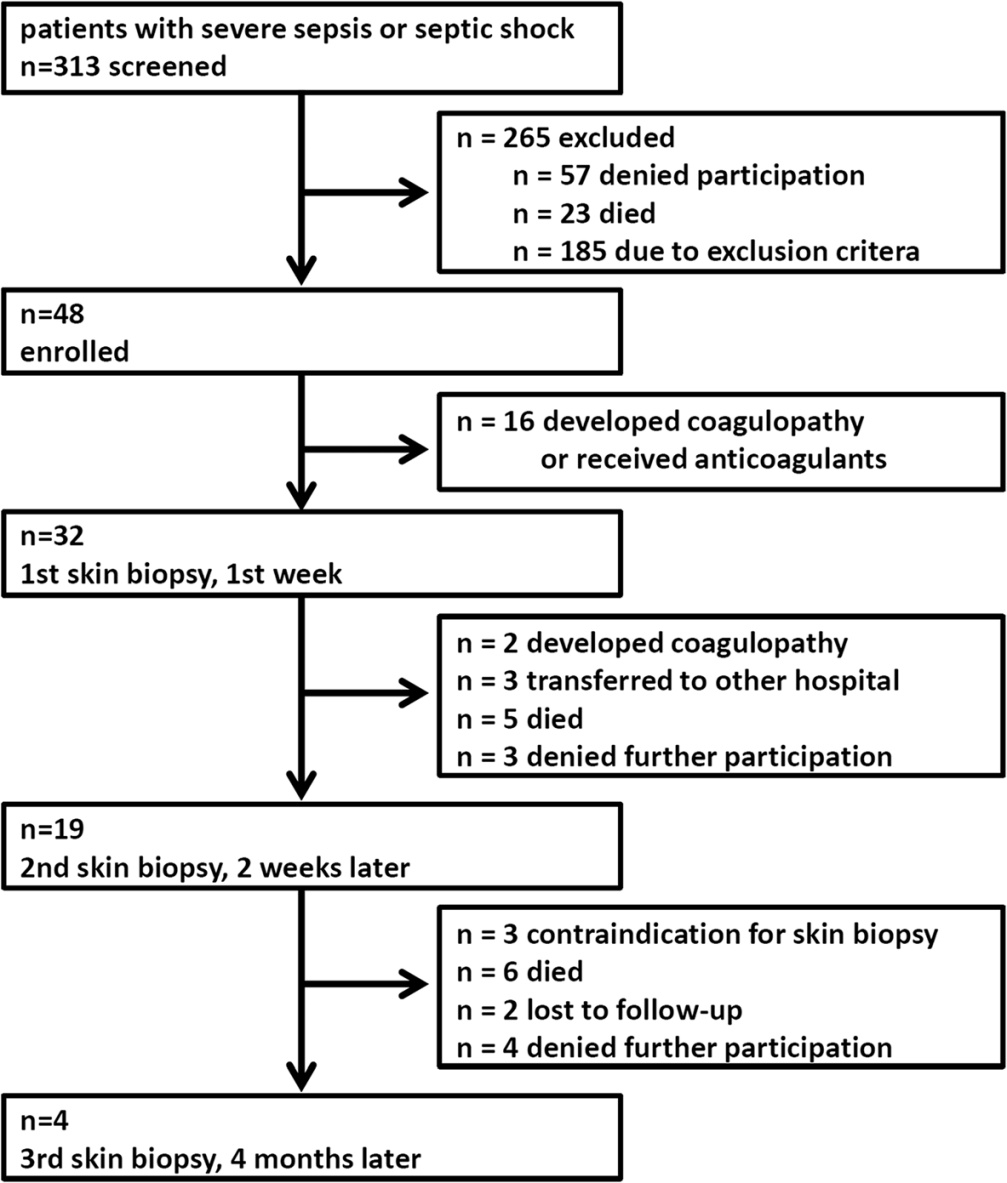
Study flow

Baseline characteristics of the patients (e.g., underlying disease, age, gender, body mass index, ICU characteristics, i.e., duration of stay, length of mechanical ventilation, etc.) were obtained from the standardized prospective sepsis registry established at Jena University Hospital [33]. Study visits were performed between days 2 and 5 after onset of severe sepsis/septic shock, and at 2 weeks and 4 months, with skin biopsies performed at the thigh and ankle. Neurological examination (including the Medical Research Council (MRC) score for muscle strength), nerve conduction studies, and sympathetic skin response were undertaken at each visit. Figure 1 summarizes patient flow and study visits.

In addition, gender- and age-matched healthy controls with a normal neurological status were visited once and skin biopsies were taken from the right ankle and thigh. The age of the matched control was not permitted to differ by more than 2 years from the age of the consecutive patient. As the controls were required to be healthy (including the older individuals) exclusion criteria were defined as: diabetes mellitus, history of neuromuscular disorders (such as polyneuropathy, myasthenia gravis, myopathy, and others), alcohol abuse, infection, history of cancer, coagulopathy, or contraindication to local anesthetics. Healthy controls were recruited in the local sport clubs to specifically include healthy people of different ages.

### Nerve conduction studies

Standardized nerve conduction studies were carried out at each visit using a portable electrophysiologic device (Synergy 15.0; Natus Europe GmbH, Planegg, Germany) with surface electrodes, according to standard procedures described in the literature [34]. The right median nerve, the right tibial nerve, the left peroneal nerve, and both sural nerves were measured. Motor and sensory nerve responses were assessed for the right median nerve, motor responses only for the tibial and fibular nerves, and sensory nerve responses for both sural nerves. This pattern was chosen for pragmatic reasons to guarantee a fast and easy electrophysiological check-up: three motor and three sensory measurements of the arm and both legs were obtained.

Sympathetic skin responses evoked by electrical stimulation of the median nerve were measured at the hands and feet as a neurophysiological equivalent of autonomic nerve function. Active surface electrodes were placed on the palmar side of the hands and the plantar side of the feet with reference electrodes on the dorsum of the hands and feet. An electrical stimulus (20–30 mA, 0.2 ms, with an irregular interval) was given to the right median nerve at the wrist. Skin potentials were recorded for a 10-s analysis period at both hands and feet. Sympathetic skin response was considered absent if there was no response after four stimuli.

Electrophysiological measurements were performed by experienced technical assistants from the neurophysiological department. Normative values for selected conduction study parameters derived from our own laboratory were used. Normative values were adjusted to age (e.g., 3.8 μV for older age), as sural nerve SNAP is often much lower in older healthy individuals.

### Skin biopsies

Skin biopsies were taken from the right ankle and thigh at each visit using a 3-mm biopsy punch and were immediately fixed in Zamboni's solution (2 % paraformaldehyde and picric acid) for approximately 24 h at 4 °C, then kept in a cryoprotective solution, and serially cut with a freezing microtome into 50-μm-thick sections. The sections were immunostained with anti-PGP 9.5 antibodies (anti protein gene product 9.5 antibody, ab72911, Abcam, Cambridge, UK) to label small nerve fibers in the skin and were counterstained with hematoxylin and eosin. Bright field microscopy for routine diagnostic purposes was used to count intraepidermal nerve fiber density (IENFD) defined by the number of stained nerve fibers crossing the basal lamina of the skin divided by the length of the epidermal surface. Image J 1.47v (National Institutes of Health, USA) was used for image processing [35].

Histological skin sections were independently analyzed by two trained raters (HA and SB) blinded for the assignment of the probe to either patient or control group. Intra-rater intraclass correlation coefficients (ICC) for estimation of IENFD were 0.977 at the ankle and 0.927 at the thigh. Inter-rater ICCs for estimation of IENFD at the ankle were 0.927 and 0.844 at the thigh. Data were reported from the first rater (HA). IEFND values were regarded as significantly reduced if they were lower than the 0.05 quantile per age span and gender according to a worldwide normative reference study [20].

### Statistics

IBM SPSS Statistics Version 19 (SPSS Inc., Chicago, IL, USA) and R Version 3.1.0 [36] were used for statistical analysis. Descriptive statistics were used to characterize demographic data, baseline characteristics of the patients, and major parameters of nerve conduction studies such as amplitudes and conduction velocity. The unpaired *t* test was used with a two-sided significance level of 5 % to compare IENFD between groups. Kaplan–Meier curves and the log-rank test were used to analyze the use of IENFD at the ankle in the first week of sepsis as a possible prognostic factor for survival.

## Results

The demographic data of our study population are presented in Table 1. 313 patients were screened, but in many cases (18 %) the patients’ legal representatives did not approve participation in the study, mainly due to the invasive character of skin biopsy. Exclusion criteria were met in 185 patients (59 %), essentially the existence of polyneuropathy, alcohol abuse, or coagulopathy. A total of 32 patients, who fulfilled the criteria for severe sepsis or septic shock, were biopsied in the first week after onset of sepsis. Healthy controls (n = 32) with normal neurological status were matched to the sepsis patients according to age and gender (19 male and 13 female individuals in both groups, mean age 66.75 years, median age 71.5 years, IQR 60.5–75.0 in the control group). Healthy controls did not have any metabolic disease (such as diabetes mellitus), neuromuscular disease, or alcohol abuse. They only had comorbidities that do not influence neuropathy (high blood pressure (n = 4), coronary heart disease (n = 2), lower back pain (n = 2), depression (n = 1), and history of basal cell carcinoma that had been completely excised and cured (n = 1)).

**Table 1:** Demographics and baseline characteristics of patients (n = 32)

Parameter Statistic	
Sociodemographic data	
Age, years, median, mean (IQR)	69, 67.41 (61.50–77.25)
Male gender, number (%)	19 (59.4)
Body mass index, kg/m^2^, mean ± SD	27.50 ± 5.04
Clinical data	
APACHE II score^a,^ mean ± SD	23.38 ± 5.73
Septic shock, number (%)	25 (78.1)
History of diabetes, number (%)	13 (40.6)
Heart failure, number (%)	5 (15.6)
Cerebrovascular disease, number (%)	15 (46.9)
Renal dysfunction, number (%)	3 (9.4)
Chronic obstructive pulmonary disease, number (%)	4 (12.5)
Liver cirrhosis, number (%)	0 (0.0)
History of cancer^b^, number (%)	2 (6.3)
Immunosuppression^c^, number (%)	4 (12.5)
Recent surgical history	
Elective surgery, number (%)	2 (6.3)
Emergency surgery, number (%)	8 (25.0)
No history of surgery, number (%)	22 (68.8)
Site of infection (multiple responses per patient possible)	
Pneumonia, number (%)	20 (62.5)
Other respiratory tract, number (%)	3 (9.4)
Intraabdominal/gastrointestinal, number (%)	6 (18.8)
Bones/soft tissue, number (%)	0 (0.0)
Surgical wound infection, number (%)	0 (0.0)
Urogenital, number (%)	1 (3.1)
Primary bacteremia, number (%)	1 (3.1)
Cardiovascular, number (%)	1 (3.1)
Others (central nervous system, thoracal, catheter, etc.), number (%)	4 (12.5)
Source of infection	
Community acquired, number (%)	9 (28.1)
Nosocomial, number (%)	23 (71.9)
Infection	
Microbiologically confirmed, number (%)	27 (84.4)
Clinical evidence, number (%)	5 (15.6)
Mortality, length of stay, mechanical ventilation, renal replacement	
ICU mortality, number (%)	2 (6.3)
Hospital mortality, number (%) (95 % CI)	5 (15.6) (6.86; 31.75)
Length of stay on ICU, calendar days, median (IQR)	24.5 (17–30.25)
Length of stay in hospital, calendar days, median (IQR)	29 (24–48.5)
Mechanical ventilation, number (%)	31 (96.9)
Days with mechanical ventilation^d^, median (IQR)	20.5 (14–29)
Renal replacement therapy, number (%)	5 (15.6)
Days with renal replacement therapy, median (IQR)	18 (18–20)

^a^Missing subscores counted as 0. ^b^At least one of leukemia, hematologic malignancy and malignant lymphoma. ^c^At least one of cytostatic therapy, radiation, immunosuppression and steroids. ^d^If not mechanically ventilated, counted as 0 days. *APACHE* Acute Physiology and Chronic Health Evaluation

All sepsis patients had CIP according to electrophysiological criteria. CIP was diagnosed, when amplitudes of compound muscle action potential and sensory nerve action potential [17, 37] were reduced in at least three nerves. Electrophysiological diagnosis of CIP was not based on pathological sural nerve potential alone as these may be notably susceptible to edema. Clinically, all patients suffered from muscle weakness (see Additional file 1). However, the majority of patients received analgo-sedation and mechanical ventilation (25 patients (78.1 %) with mechanical ventilation at first visit). None of the patients were exposed to high-dose steroids (exclusion criteria of the study) or neuromuscular blocking agents. No patient suffered from HIV. All patients received passive or active physiotherapy.

Nerve conduction studies displayed changes typical of axonal damage during the first week after onset of sepsis. Reductions in amplitudes of motor and sensory action potential were more pronounced than reductions in conduction velocity. Electrophysiological parameters did not change significantly over the time course of sepsis (see box plots of amplitudes of action potential and conduction velocity in Additional file 2). The sympathetic skin response at the hands and feet was lost in all patients.

**Fig. 2:**
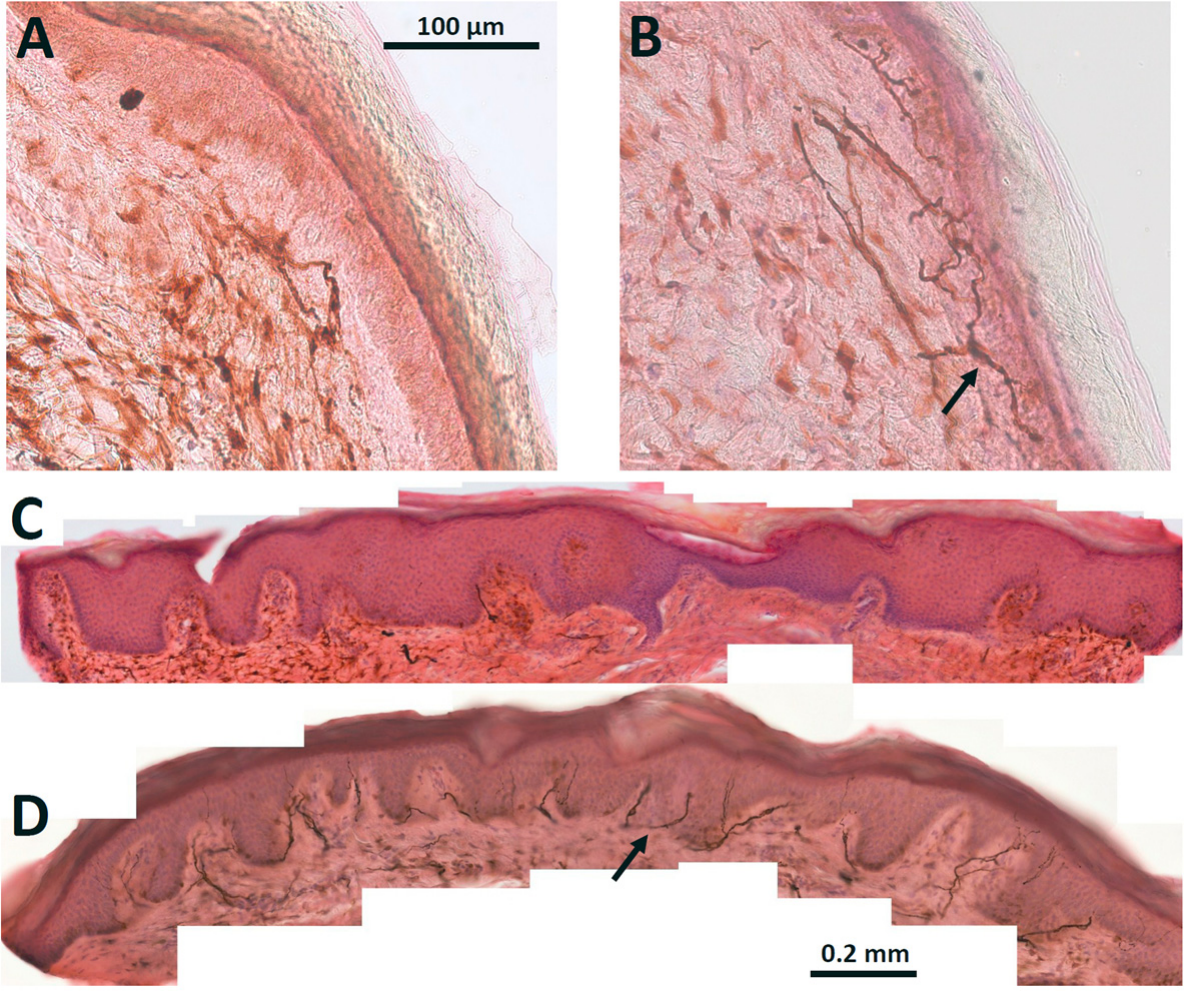
Example of histology of skin biopsies in septic patients (**a** and **c**) and normal controls (**b** and **d**). Intraepidermal nerve fiber density is considerably decreased in septic patients. *Arrows* show examples of nerve fibers

**Fig. 3:**
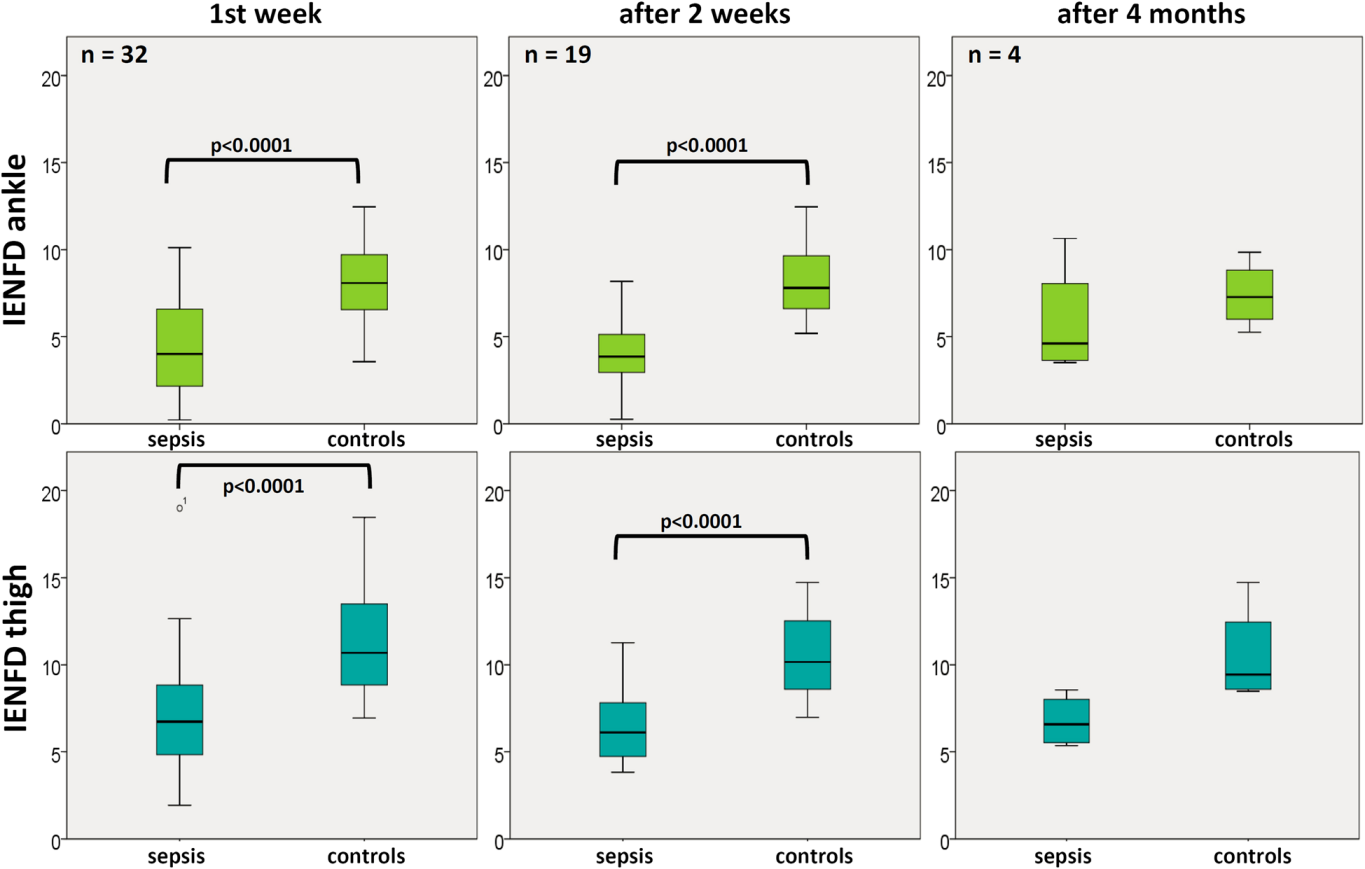
Intraepidermal nerve fiber density (*IENFD*) in patients (*sepsis*) and controls over time. IENFD in patients with sepsis are significantly decreased in comparison to age- and gender-matched controls

Skin biopsies (Fig. 2) revealed a considerable decrease in small nerve fibers in the septic patients early in the course of disease. Eleven of the patients (34 %) showed IEFND values lower than the 0.05 quantile per age span and gender according to a worldwide normative reference study [20] during the first week of severe sepsis or septic shock. Figure 3 shows the boxplots for intraepidermal nerve fiber density at the ankle and thigh of the patients in comparison to the controls. IENFD at the ankle and thigh were significantly decreased (p <0.0001) in patients compared to age- and gender-matched controls during the first week of sepsis (n = 32) and 2 weeks later (n = 19). After 4 months, four patients were subjected to a third skin biopsy. In these patients IEFND was still reduced. The results were not statistically significant (*p* = 0.452 at the ankle, *p* = 0.076 at the thigh) due to the small sample size. The main reasons (Fig. 1) for not obtaining follow-up skin biopsies were death and the development of contraindications to skin biopsy, such as anticoagulation or coagulopathy.

The ratio between IENFD at the ankle and thigh did not differ between patients and controls (*p* = 0.103 at visit 1, *p* = 0.113 at visit 2, and *p* = 0.8 at visit 3). This ratio is an indicator of length-dependent nerve damage; it is considerably increased if distal nerve fibers (at the ankle) are impaired to a much greater extent than proximal nerve fibers (at the thigh).

At the first visit only 7 of the 32 patients were evaluated for sensory symptoms such as sensory loss or dysesthesia. Mean pallesthesia was 3.7 (on a scale between 0 (pallanaesthesia) and 8 (normal pallaesthesia)) with standard deviation of 3.3 measured by the use of a tuning fork placed over the lateral malleolus. At visit 2, there were 10 out of 19 patients evaluated: only 1 of the 10 patients complained of sensory symptoms. The four patients examined after 4 months had mean pallesthesia of 5.6 with standard deviation of 1.5. Three of these patients complained of sensory symptoms.

**Fig. 4:**
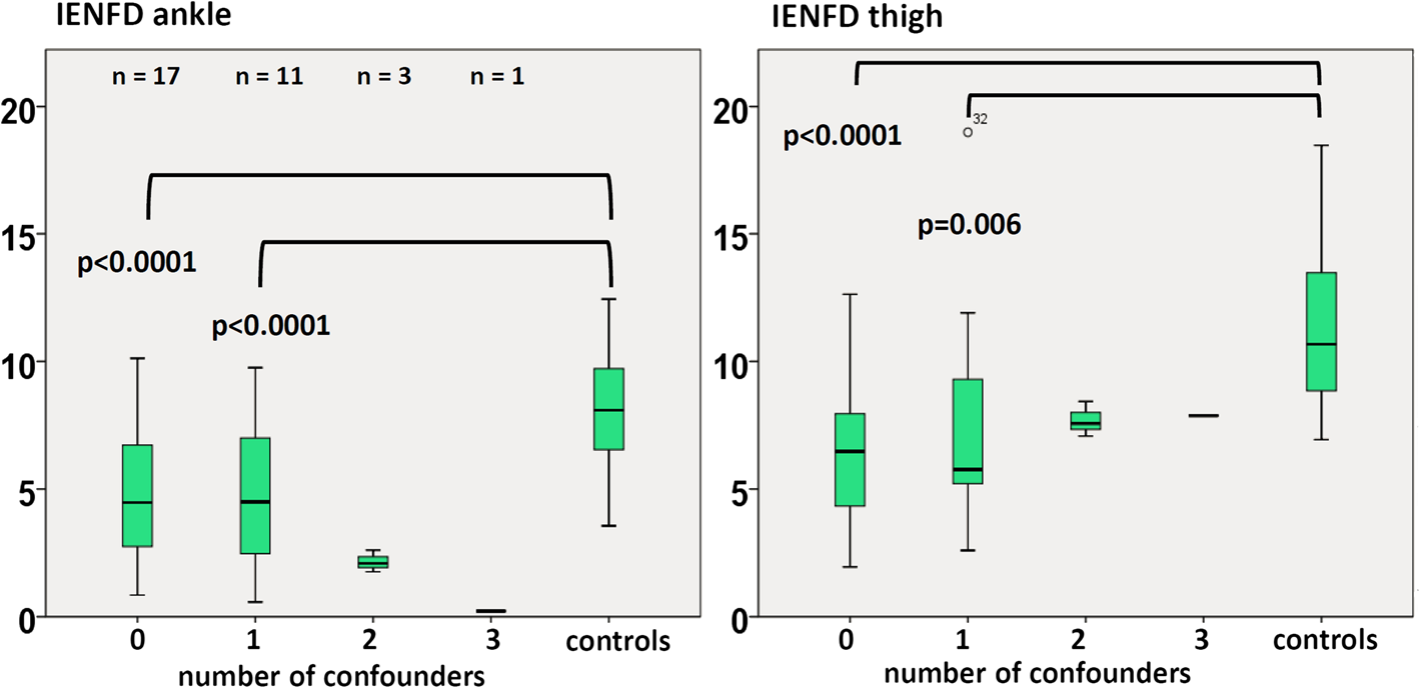
Intraepidermal nerve fiber density (*IENFD*) over time in patients grouped according to number of confounding comorbidities for neuropathy. In patients without any confounding comorbidities (n = 17) there was significant loss of IENFD at ankle and thigh in comparison to the healthy controls

In addition, patients with sepsis were grouped according to the number of confounding co-morbidities for neuropathy (especially diabetes, history of cancer and chronic kidney disease, Table 1). Alcohol abuse was an exclusion criterion. IENFD at the ankle and thigh was significantly reduced in the 17 patients without confounding comorbidities in comparison to the healthy controls (Fig. 4).

**Fig. 5:**
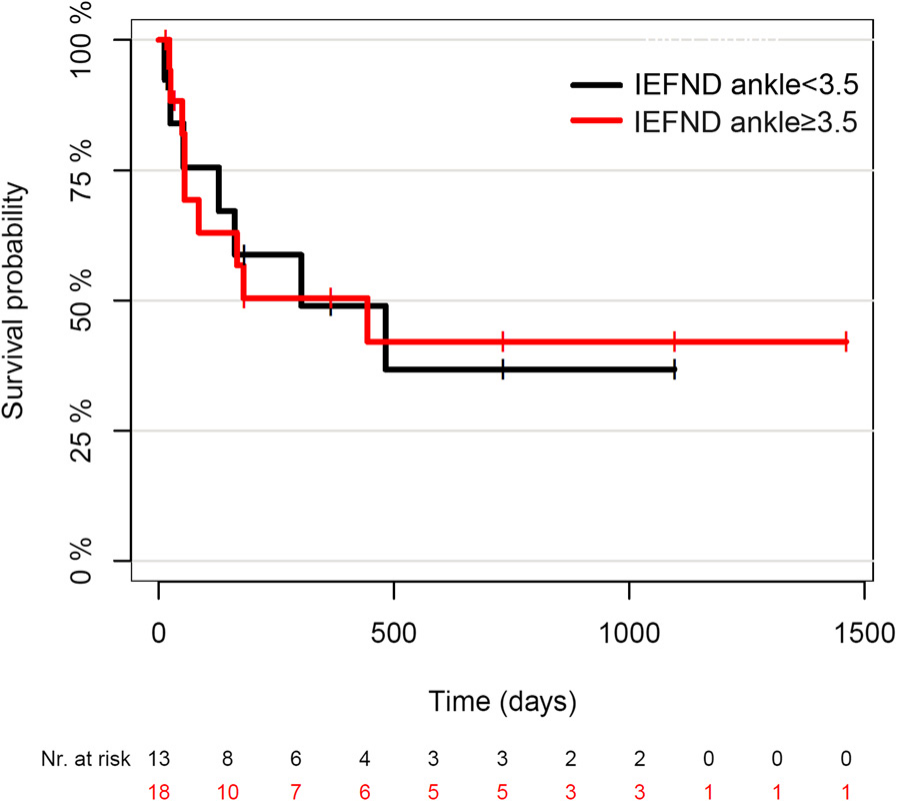
Kaplan–Meier curves for survival time in the patients grouped according to intraepidermal nerve fiber density. Log-rank test 0.927. *Nr* number

Patients were divided into two groups comprising those with IEFND below, and those with IEFND >3.5 fibers/mm of skin, to test whether in the acute phase of sepsis IENFD at the ankle is a prognostic marker for survival. On Kaplan–Meier curves there were no significant differences in survival time in the two groups (*p* = 0.927, Fig. 5). Median survival time was 444 days for patients with an IENFD <3.5 fibers/mm and 303 days for patients with an IENFD >3.5 fibers/mm.

## Discussion

Skin biopsy has become a valuable tool in the evaluation of patients with small nerve fiber pathological changes [21]. Skin biopsy represents a well-established, safe, easy, and inexpensive method to quantify small sensory nerve fibers and autonomic nerve fibers in the skin [18]. In contrast, nerve conduction studies are not able to evaluate the function of small nerve fibers. Damage to small nerve fibers has recently been demonstrated in small samples of critically ill patients [29, 30]. Small fiber pathological change may play a role in the pathophysiology of sepsis, e.g., in neuropathic pain syndromes, which often become apparent after recovery from severe illness and after discharge from the ICU [23]. Although sensory problems like neuropathic pain, stocking/glove sensory loss, numbness and pallhypesthesia were evaluated in this study, the majority of patients were not able to report these reliably in the acute phase of sepsis. As neuropathic pain is a problem in the long-term follow up of patients with sepsis, a study addressing this specific issue has to be planned using longer periods of follow up.

In addition, autonomic dysregulation plays a significant role in the acute phase of severe sepsis [24]. Lately, the occurrence of CIP has been related to decreased heart rate variability [25]. As autonomic dysregulation is associated with increased long-term mortality [26, 27], impairment of small nerve fibers may have an impact on the prognosis of patients with sepsis.

In our study all patients suffered from CIP according to electrophysiological criteria. All patients had loss of sympathetic skin response in the hands and feet. The latter observation may also be influenced by catecholamine therapy [38] and can therefore not be regarded as a secure electrophysiological sign of autonomic neuropathy.

A major finding of this study is the early decrease in IEFND during the first week of severe sepsis and septic shock. In the present study, the ratio of IENFD at the ankle (distal) and thigh (proximal) of patients and controls were not length-dependent. Length-dependent axonal degeneration (the so-called dying-back phenomenon) results in a typically distal-to-proximal gradient of axonal degeneration with symmetrical and distally pronounced neuropathic symptoms.

The reduction in intraepidermal nerve fibers in our patients, who received the first biopsies on median day 4 after onset of sepsis, was similar to that observed by Latronico et al. [29], who performed skin biopsies on median ICU day 22. Skorna et al. [30] performed skin biopsies on the admission to the ICU and 10–14 days later. They demonstrated that IENFD decreased in 8 of 13 patients (72.7 %) after the admission to the ICU.

Although we excluded patients with known neuromuscular disorders such as polyneuropathy, one has to take into consideration that 40.6 % of our patients suffered from diabetes, which is a risk factor for peripheral nerve damage. However, subgroup analysis showed that the 17 patients with sepsis who did not have confounding comorbidities for neuropathy also had decreased IENFD similar to those patients with one confounder for neuropathy, such as diabetes, chronic kidney disease, or history of cancer. To overcome this potential source of error, future studies should be based on patients admitted to the ICU with and without sepsis to study sepsis-specific neuropathy. All examinations of neuromuscular disturbance in sepsis have their own limitations. A reliable estimation of muscle strength in non-alert and non-awake patients is generally not possible. In many cases temporal interruption of sedative drugs is not possible in hemodynamically instable patients. Therefore, the MRC scores often cannot be reliably measured in the acute phase of sepsis.

Amplitudes of compound motor action potential and sensory nerve action potential in nerve conduction studies (NCS) may also be reduced due to edema, which is often a problem in sepsis. Moreover, NCS findings do not exclude myopathy. As CIP and CIM often occur together [39, 40], and muscle biopsies or direct muscle stimulation were not performed to distinguish CIP from CIM in this study, patients may also suffer from CIM. The difference is also important with respect to the prognosis, because the consequences of CIP last much longer than those of CIM [41], in terms of persistent muscle weakness and the development of chronic pain syndromes [29, 42]. In addition, repetitive nerve stimulation was not performed to rule out myasthenia-type abnormalities. Skin biopsy allows direct allocation of the damage to the peripheral nerves. It, therefore, may facilitate the differentiation between CIP and CIM. However, the study was not designed to address this research question. Although skin biopsy is an invasive method it causes only minimal pain using local anesthesia. The histological work-up and analysis of the skin biopsy takes some days, so that the result of the skin biopsy is not directly available at the bedside, which is a pragmatic restriction of the method.

Many of the legal representatives of the patients rejected the participation of the patient in the study because they feared the invasive character of skin biopsy. In addition, the study had a high drop-out rate of patients over time due to high mortality rates and contraindications against skin biopsy (such as coagulopathy or anticoagulation). Therefore, the initial target number of patients (n = 200) as designated in the study protocol [31] was not reached although over 300 patients were screened. However, the extent the decrease in IENFD was much larger than expected and the results were statistically significant based on considerably fewer patients than initially scheduled.

IEFND values did not change considerably over a longer time course. In addition, the time course of decrease of IEFND was similar to the time course of nerve conduction study abnormalities. This is consistent with a predominant axonal type of neuropathy with slow regeneration of the nerves (about 1 mm per day) which requires long-lasting rehabilitation. However, there are extreme biases related to the four patients who underwent repeat biopsies at 4 months. These biases unfortunately question the value of these results. Therefore, we cannot draw reliable conclusions from these results.

We also tested for IENFD in the first week of sepsis as a prognostic biomarker for survival, because autonomic neuropathy may cause autonomic dysregulation and leads to increased mortality rates [25, 27]. In a follow-up analysis of survival among our patients using the prospective sepsis registry, a clear prognostic value of IENFD at the ankle in the first week of sepsis was not be observed. The cutoff value of 3.5 fibers/mm was arbitrarily chosen for practical reasons although IENFD normative values are age- and sex-dependent [20]. All 11 patients with an IEFND <0.05 quantiles per age span and gender were included in the group with an IEFND <3.5 fibers/mm and their survival rate did not differ from that in the other groups. However, the sample size may have been too small to differentiate survivors from non-survivors. A larger study would be needed to definitely conclude that IEFND may not be suitable as a prognostic parameter for survival.

## Conclusions

The density of small sensory nerve fibers in the skin is reduced early in the course of sepsis. This reduction does not depend on the length of the axons. The decrease in intraepidermal nerve fibers does not considerably change over time in the acute phase of sepsis; it is not a good predictor for survival.

## Key messages

- Skin biopsy detects impairment of small sensory nerve fibers early in the course of sepsis
- It is not suitable as a prognostic marker for survival
- Intraepidermal nerve fiber density does not change considerably over the first weeks of sepsis

## Additional files

Additional file 1: Medical Research Council (*MRC*) scores of muscle weakness over time. MRC scores of muscle strength were quantified bilaterally in the arms and legs. Figure shows examples of different proximal and distal movements of the limbs and MRC sum score. Note that the majority of patients could not reliably be evaluated due to analgo-sedation (2 patients with Richmond Agitation Sedation Scale (*RASS*) < -1 in the second week and mechanical ventilation (78 % of patients (n = 25) in the first week and 50 % (n = 9) in the second week), so that muscle strength could only be evaluated in 7 patients in the first week and 10 patients in the second week. In the patients examined after 4 months there was a considerable improvement in muscle strength, but muscle weakness was still detectable. Muscle weakness was always symmetrical and affected both proximal and distal muscle groups. (PDF 533 kb) Additional file 2: Nerve conduction studies. Amplitudes of compound potentials in motor and sensory nerves are considerably decreased, and conduction velocity is marginally impaired. Note that nerve conduction studies detect critical illness polyneuropathy early in the course of sepsis, but amplitudes do not change considerably over the course of the disease. *Horizontal lines* show the normative values of the measurements. (PDF 340 kb)

## Abbreviations

CIM: critical illness myopathy
CIP: critical illness polyneuropathy
ICU: intensive care unit
ICUAW: ICU-acquired weakness
IENFD: intraepidermal nerve fiber density
MRC: Medical Research Council
RASS: Richmond Agitation Sedation Scale
SNAP: sensory nerve action potential
