# Supplementary material for: The impairment of small nerve fibers in severe sepsis and septic shock
Source: Crit Care. 2016 Mar 15;20:64. doi: 10.1186/s13054-016-1241-5 (PMC4793743; doi:10.1186/s13054-016-1241-5)
Supplement: Additional file 1: — Medical Research Council (MRC) scores of muscle weakness over time. MRC scores of muscle strength were quantified bilaterally in the arms and legs. Figure shows examples of different proximal and distal movements of the limbs and MRC sum score. Note that the majority of patients could not reliably be evaluated due to analgo-sedation (2 patients with Richmond Agitation Sedation Scale (RASS) < -1 in the second week and mechanical ventilation (78 % of patients (n = 25) in the first week and 50 % (n = 9) in the second week), so that muscle strength could only be evaluated in 7 patients in the first week and 10 patients in the second week. In the patients examined after 4 months there was a considerable improvement in muscle strength, but muscle weakness was still detectable. Muscle weakness was always symmetrical and affected both proximal and distal muscle groups. (PDF 533 kb) [file 13054_2016_1241_MOESM1_ESM.pdf]

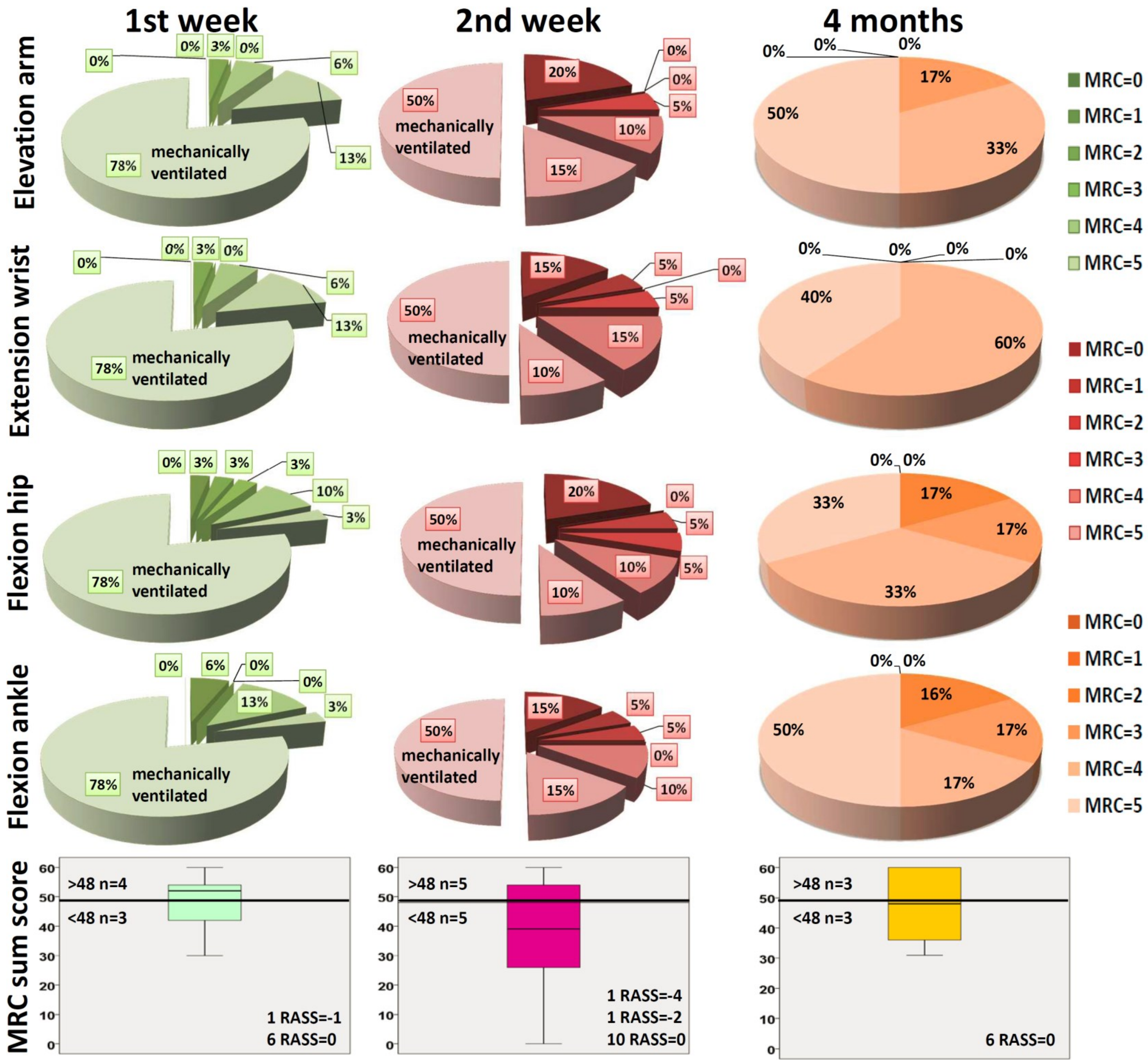

Appendix 1: MRC (Medical Research Council) scores of muscle weakness over time. MRC scores of muscle strength were quantified bilaterally in arms and legs. The figure shows examples of different proximal and distal movements of the limbs and MRC sum score. Note that the majority of patients could not reliably be evaluated due to analgosedation (2 patients with RASS < -1 in the second week, RASS = Richmond Agitation Sedation Scale) and mechanical ventilation (78% of patients (n=25) in the first week and 50% (n=9) in the second week), so that muscle strength in only 7 patients in the first week and 10 patients in the second week could be evaluated. In the patients examined after 4 months a considerable improvement of muscle strength could be found, but muscle weakness was still detectable. Muscle weakness always was symmetrical and affected both proximal and distal muscle groups.
