# Supplementary material for: The impairment of small nerve fibers in severe sepsis and septic shock
Source: Crit Care. 2016 Mar 15;20:64. doi: 10.1186/s13054-016-1241-5 (PMC4793743; doi:10.1186/s13054-016-1241-5)
Supplement: Additional file 2: — Nerve conduction studies. Amplitudes of compound potentials in motor and sensory nerves are considerably decreased, and conduction velocity is marginally impaired. Note that nerve conduction studies detect critical illness polyneuropathy early in the course of sepsis, but amplitudes do not change considerably over the course of the disease. Horizontal lines show the normative values of the measurements. (PDF 340 kb) [file 13054_2016_1241_MOESM2_ESM.pdf]

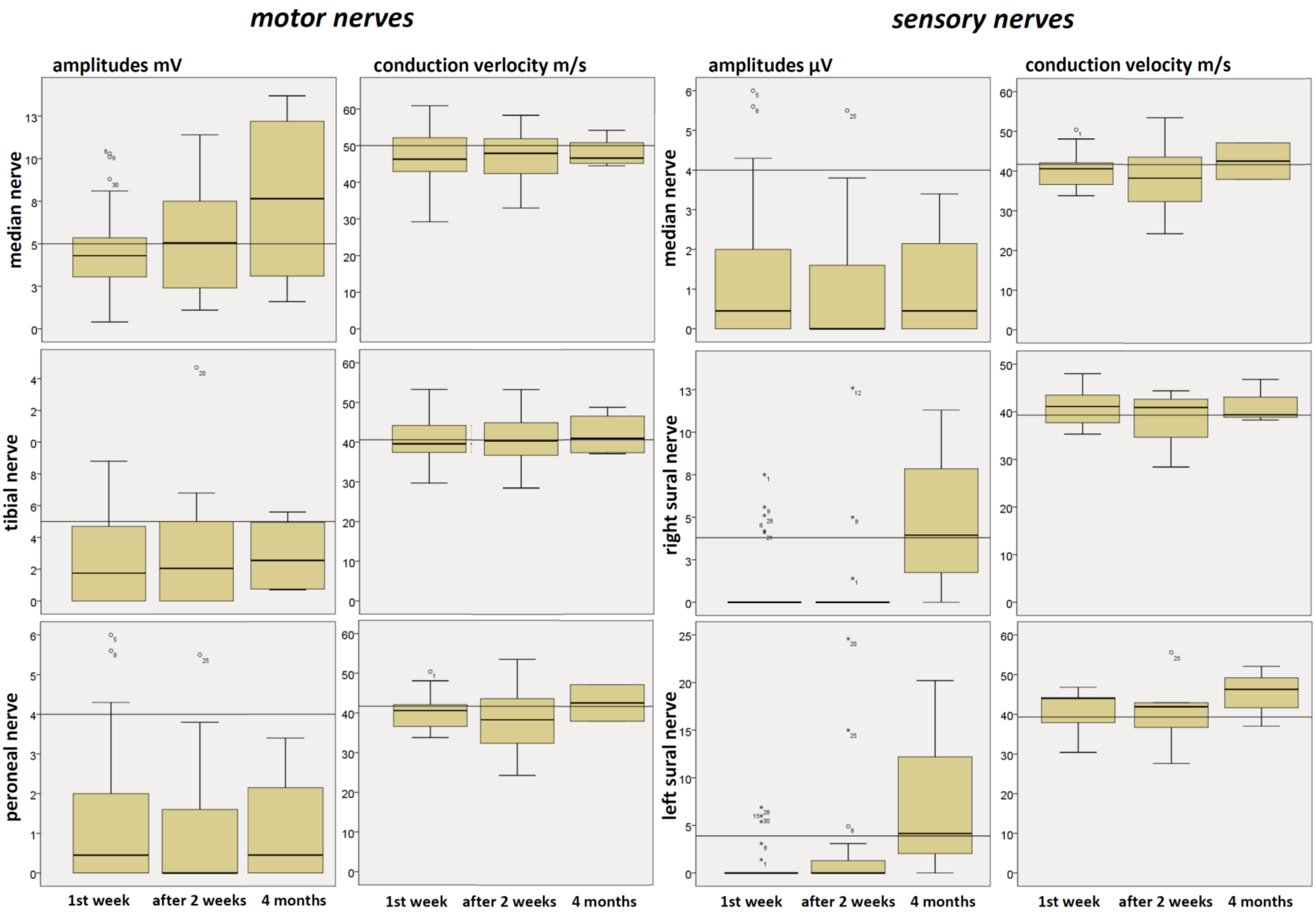

Appendix 2: Nerve conduction studies. Amplitudes of compound potentials in motor and sensory nerves are considerably decreased, while conduction velocity is marginally impaired. Note that nerve conduction studies are able to detect CIP early in the course of sepsis, but amplitudes do not change considerably over the course of the disease. Horizontal lines show the normative values of the measurements.
